# The Role of ABA in the Interaction between Citrus Fruit and *Penicillium digitatum*

**DOI:** 10.3390/ijms232415796

**Published:** 2022-12-13

**Authors:** María T. Lafuente, Luis González-Candelas

**Affiliations:** Department of Food Biotechnology, Instituto de Agroquímica y Tecnología de Alimentos (IATA-CSIC), Agustín Escardino 7, Paterna, 46980 Valencia, Spain

**Keywords:** abscisic acid (ABA), fungal disease, green mold, postharvest rots, transcriptomic profiling

## Abstract

Abscisic acid (ABA) protects citrus fruit against *Penicillium digitatum* infection. The global mechanisms involved in the role of ABA in the *P. digitatum*–citrus fruit interaction are unknown. Here, we determine the transcriptome differences between the Navelate (*Citrus sinensis* (L.) Osbeck) orange and its ABA-deficient mutant Pinalate, which is less resistant to infection. Low ABA levels may affect both the constitutive mechanisms that protect citrus fruit against *P. digitatum* and early responses to infection. The repression of terpenoid, phenylpropanoid and glutation metabolism; of oxidation–reduction processes; and of processes related to the defense response to fungus and plant hormone signal transduction may be one part of the constitutive defense reduced in the mutant against *P. digitatum*. Our results also provide potential targets for developing *P. digitatum*-citrus fruit-resistant varieties. Of those up-regulated by ABA, a thaumatin protein and a bifunctional inhibitor/LTP, which are relevant in plant immunity, were particularly remarkable. It is also worth highlighting *chlorophyllase 1* (*CLH1*), induced by infection in Pinalate, and the *OXS3* gene, which was down-regulated by ABA, because the absence of OXS3 activates ABA-responsive genes in plants.

## 1. Introduction

Major food waste and economic losses are due to disease development caused by phytopathogenic fungi during postharvest handling and storage [1]. Citrus fruit are one of the most important horticultural crops worldwide. The necrotrophic fungus *Penicillium digitatum* (Pers.:Fr.) Sacc. is this crop’s main postharvest pathogen, which causes green mold disease. The control of this fungus is carried out with synthetic fungicides, which increasingly have major drawbacks due to the emergence of fungal strains that resist commonly-employed fungicides; leading to consumer concerns about problems related to health and environmental pollution and the requirements from importing markets of ‘zero residue’ produce. Finding alternatives to these fungicides remains a priority [2]. Therefore, it is necessary to acquire better knowledge about the mechanisms that participate in citrus fruits’ resistance to *P. digitatum*.

Plant hormones play important roles in the susceptibility of plants and fruit to infection by pathogens [1,3,4]. These hormones may be induced upon fungi attack by infected cells and act as signaling molecules by targeting mechanisms in surrounding cells that positively or negatively affect fungal spread [5,6]. Studies that have focused on the involvement of the hormone abscisic acid (ABA) in fruit crops’ susceptibility to phytopathogenic fungi are scarce. However, it is known that ABA enhances pathogen susceptibility in some fruit such as tomato [7,8] or pepper [9], but favors pathogen resistance in grapes and citrus fruit [10,11]. The different effects of ABA depend on the hormone-induced mechanisms in a specific plant/crop–pathogen interaction.

We recently showed that both citrus fruit and *P. digitatum* produce ABA, that fungal ABA is not a primary virulence factor, and that fruit ABA plays a protective role against *P. digitatum* infection [11]. This was demonstrated by comparing the susceptibility of Navelate (*Citrus sinensis* (L.) Osbeck) orange and its natural yellow mutant Pinalate, which contains very low ABA levels [12], to fruit infection, and assessing how such this susceptibility was altered by exogenous ABA [11]. In this work, we also examined the effect of three inhibitors of the synthesis of the ABA hormone: (1) norflurazon, which acts in the initial steps of its synthesis by inhibiting phytoene desaturase; (2) nordihydroguaiaretic acid (NDGA), which inhibits a key enzyme (9-cis epoxycarotenoid dioxygenase) in ABA biosynthesis in oranges; and (3) tungstate, which inhibits the last step in ABA biosynthesis. NDGA and tungstate reduced the in vitro growth of *P. digitatum* and, therefore, its viability to infect fruit, while norflurazon damaged the fruit peel, which should increase disease incidence [11]. Therefore, the above-mentioned inhibitors could not be used in pharmacological experiments, but the Pinalate mutant is a valuable tool to further investigate the role of ABA in citrus fruit infection. The availability of artificially-generated mutants is uncommon in woody plants and, to our knowledge, there is no ABA knockout mutant in citrus fruit. Despite Pinalate fruit being a spontaneous rather than a knockout mutant [12,13], and its partial ABA insensitivity [14], access to this mutant has been of particular scientific interest to understand the role of this hormone in both peel ripening [15] and dehydration stress [16,17] in citrus fruit, and to characterize the Citrus ABA signalosome during both physiological processes [14].

To the best of our knowledge, there is only one report that has focused on studying the mechanisms involved in the beneficial effect of ABA for increasing citrus fruit resistance to *P. digitatum*, which centered on the interrelation between ABA and specific phospholipases [18]. In the present study, we conducted a systematic comprehensive study to better understand the interrelationship between ABA and the global mechanisms involved in citrus fruits’ natural defense against *P. digitatum*, and the mechanisms regulated by ABA in the citrus fruit infected by this phytopathogenic fungus. To this end, we focused on the transcriptomic differences between the flavedo (outer part of peel) of Navelate and Pinalate oranges upon fruit harvest, and, before inoculating them with the pathogen, examined any changes induced by the fungus in the flavedo of both cultivars at the transcriptomic level. Moreover, and considering: (1) that applying ABA 1 mM reduces disease incidence in Pinalate orange but has a negligible effect on infection in Navelate, which already has very high ABA content [11]; and (2) previous results from our group [11,17] indicating that endogenous levels of the phytohormone are sufficient to trigger cellular processes coping with biotic and abiotic stresses, irrespective of the fruit maturity stage and, therefore, that the consequences of such stresses are not modified by applying ABA; we restricted the study to the effect of exogenous ABA on the mutant’s response to *P. digitatum* infection. Early response genes are key for perceiving and amplifying stress signals and for targeting downstream gene expression [4]. Moreover, previous research into citrus fruit suggest that the contribution of fruit genes prevails over that of the fungus in early infection stages [19]. Therefore, we concentrated on determining the initial defense responses produced by *P. digitatum* in both orange cultivars, which occurred before disease and tissue degradation development.

## 2. Results

### 2.1. Differences in the Susceptibility of Navelate and Pinalate Oranges to P. digitatum Infection

**Figure 1:**
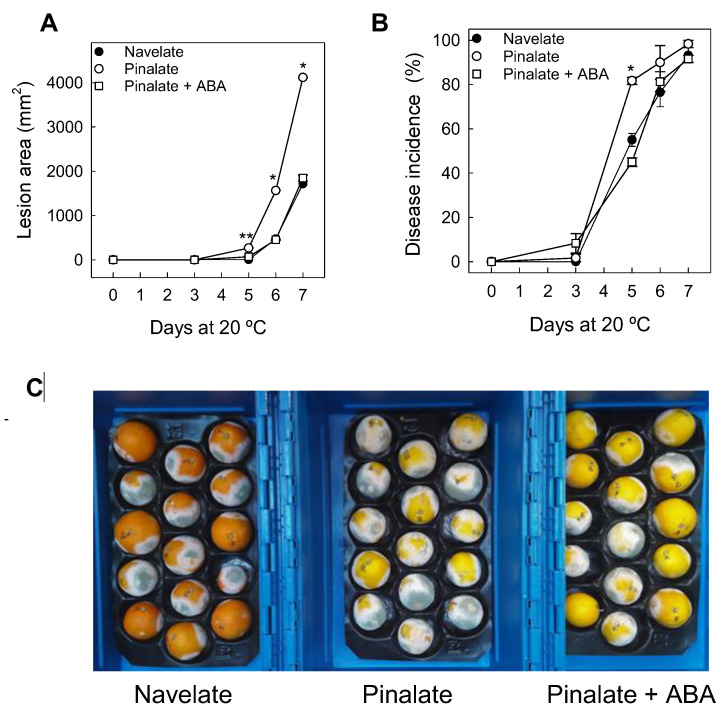
Differences in the susceptibility of Pinalate and Navelate fruit to infection by *P. digitatum*. (**A**) Changes in the lesion area of the macerated zone of Pinalate (○) and Navelate fruit (●) and of the Pinalate fruit treated with ABA 1 mM (□); (**B**) Changes in the percentage of decay in the same samples; (**C**) Picture of Navelate, Pinalate, and the ABA-treated Pinalate fruit infected (8 dpi) with *P. digitatum*. The fruit were inoculated with *P. digitatum* (10^4^ conidia mL^−1^) at a depth of 4 mm. The error interval indicates the standard error of the estimated mean value. * denotes no significant differences (*p* ≤ 0.05) between the Navelate and Pinalate fruit treated with ABA for the same storage period, but their mean values were significantly lower than those of the Pinalate fruit. ** represents significant differences (*p* ≤ 0.05) between the parental and both the mutant fruit treated or not with ABA.

Pinalate fruit showed negligible initial infection symptoms by day 3 (Figure 1A), when disease incidence was very low (Figure 1B). Thereafter, both disease incidence and severity increased. The trend of the changes in the lesion area caused by infection and the percentage of the ABA-treated Pinalate fruit showing disease were generally lower and similar to those of the parental fruit. By day 6, most fruit were already infected and the mean lesion area caused by the fungus in Navelate and in the ABA-treated Pinalate fruit was about 2.4-fold lower than in Pinalate. Accordingly, mycelium and sporulation development were more marked in the mutant, and this effect was attenuated by exogenous ABA (Figure 1C). Considering the time course of infection shown in Figure 1, and that early host responses are major determinants for the evolution of disease progression, we selected a sampling time of 1 d post-inoculation (dpi) to compare the transcriptional changes induced by *P. digitatum* in the flavedo of both cultivars and in the ABA-treated mutant.

### 2.2. Effect of Infection with P. digitatum on the Comparative ABA Levels and Transcriptional Profiling between Navelate and Pinalate

**Figure 2:**
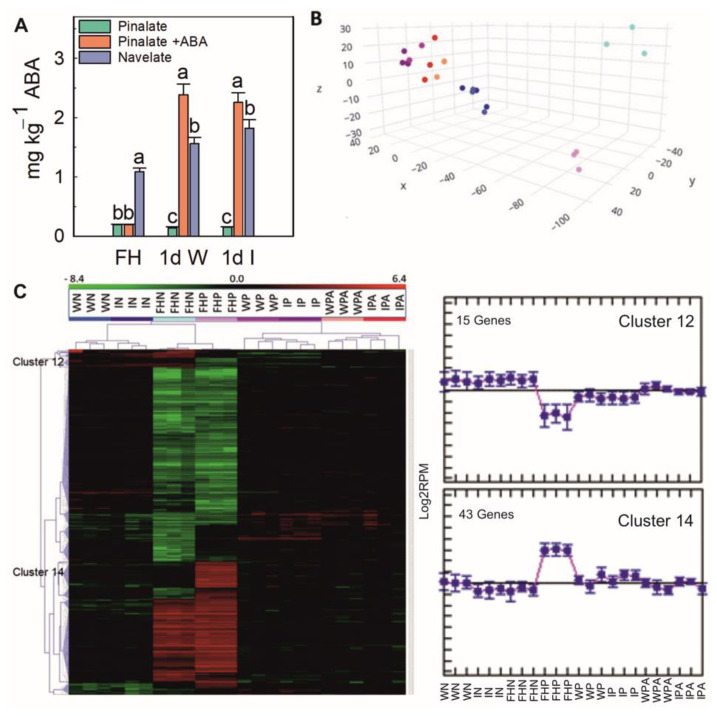
ABA content in both the freshlyharvested (FH) fruit and wounded (1d W) and infected (1d I) samples taken at 1 dpi. Different letters mean significant differences (*p* ≤ 0.05) between Navelate fruit and its mutant Pinalate, treated or not with ABA, for the same storage period. (**A**) The Principal Component Analysis (PCA) (**B**) and the heatmap of the hierarchical cluster analysis (HCA) (**C**) of the expressed genes according to the RNA-Seq analysis. The DEGs meeting a cutoff of STEDV > 0.6 and |Log_2_ FoldChange| ≥ 1 were considered in the HCA for all the conditions. The colors used in the PCA for each sample are the same as those employed for the same samples on the heatmap. FHN: freshly-harvested Navelate; WN: wounded Navelate at 1 dpi; IN: infected Navelate at 1 dpi; FHP: freshly-harvested Pinalate; WP: wounded Pinalate at 1 dpi; IP: infected Pinalate at 1 dpi; WPA: wounded Pinalate treated with ABA at 1 dpi; IPA: infected Pinalate treated with ABA at 1 dpi. The colors on the heatmap represent the median centered Log_2_(RPM) expression values for each condition and change from dark red (induction) to light green (repression). Clusters 12 and 14 are highlighted in Figure 1C. The samples on the *X*-axis follow the same order as on the heatmap. Three biological replicates from each condition were used for all the analyses. The error interval indicates the standard error of the estimated mean value.

Under the experimental conditions of the present work, the ABA content in the flavedo of Navelate fruit was 5.6-fold higher than that of Pinalate at fruit harvest (Figure 2A). This difference was slightly larger by 1 dpi in the wounded and infected fruit but was counteracted by applying ABA (Figure 2A). In light of these results and the different susceptibility of both cultivars to *P. digitatum* (Figure 1), we examined the differences between their transcriptomes at fruit harvest and in their early responses to infection, and how they can be modified by applying ABA to Pinalate.

The results of both the principal components analysis (PCA) (Figure 2B) and the hierarchical clustering analysis (HCA) (Figure 2C) showed that the repeatability of the RNA-Seq data across the three biological replicates of each sample (condition) was good, because the transcriptional profiles of all the replicates were generally grouped together. In agreement with this finding, a good correlation (r^2^ = 0.805 *p* ≤ 0.05) (Figure S1) between the results obtained from the RNA-Seq and the RT-qPCR validation analyses with the selected genes (Table S1) was found. Therefore, the RNA-Seq data could be reliably used in subsequent analyses. The PCA and HCA analyses also revealed that wounding and infection induced relevant changes in the gene expression pattern in relation to the freshly-harvested (FH) fruit of the same cultivar, which was more marked in Pinalate. This effect was attenuated by applying ABA. The differences found between the wounding and infection of the same cultivar were less marked, but relevant when comparing both cultivars at fruit harvest. This was in concordance with the gene clustering of the 615 DEGs represented on the heatmap (Figure 2C). These DEGs were grouped into 19 clusters (Figure 2C and Figure S2), of which clusters 12 and 14 with 15 and 43 genes, respectively, are worth highlighting. The DEGs expression in cluster 12 was lower in the FH Pinalate fruit than for any other condition examined in either Navelate or Pinalate fruit, while the DEGs in cluster 14 were more expressed in Pinalate at harvest than for any other condition. The functional categorization of the DEGs in cluster 12 suggest that the mutant may have poor defense responses (Table 1).

As shown in Table S2, they involve a pathogenesis-related protein (PR4), a serine protease inhibitor or the genes encoding the HXXXD-type acyl-transferase family protein. However, these responses could have been activated in Pinalate fruit in response to wounding or infection (Table S3). Likewise, the TopGO analysis of the DEGs cluster 14 highlighted that ubiquitination, phospholipid transport and protein binding (Table 1), which mostly included the DEGs related to disease resistance or encoding receptor-like proteins (RLPs) (Table S2), showed a higher expression in the FH Pinalate fruit in relation to any other studied condition—including wounding and infection in both Navelate and Pinalate fruit irrespective of ABA treatment.

**Figure 3:**
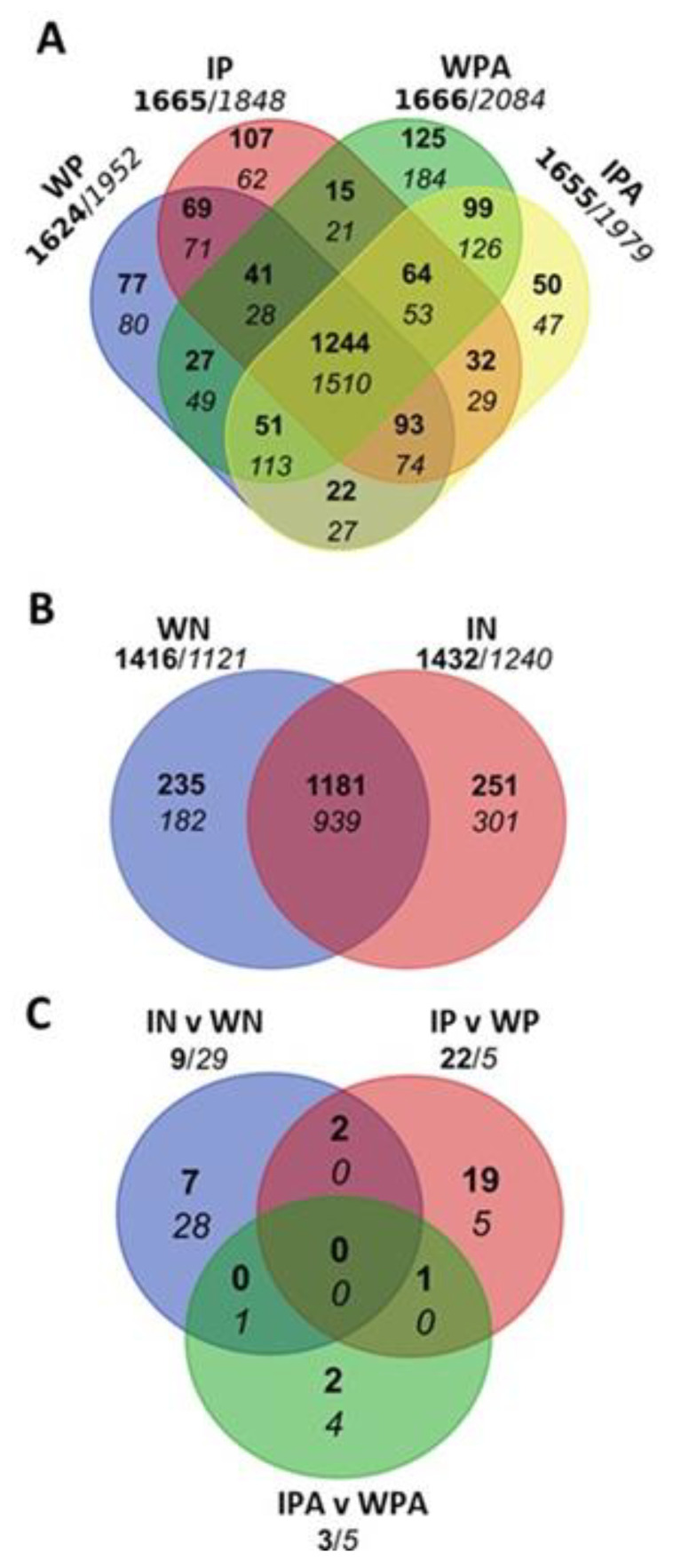
Venn diagrams of the up-(bold numbers) and down-regulated (regular numbers) genes in several comparisons (DEG, edgeR, BH *p*-value adjustment α ≤ 0.05). All the induced and repressed DEGs met a cutoff of |Log_2_ FoldChange| ≥ 1. (**A**) The number of the up- and down-regulated genes of the infected Pinalate (IP) fruit and its respective wounded controls (WP), plus those DEGS of the infected Pinalate fruit treated with ABA (IPA) and their control (WPA), compared to the freshly-harvested (FH) Pinalate fruit; (**B**) The number of the up- and down-regulated genes in the wounded (control) (WN) and infected Navelate (IN) fruit compared to the FH Navelate fruit; (**C**) The number of the up- and down-regulated genes by infection in relation to wounding (control) in the Pinalate, Pinalate + ABA and Navelate samples. The numbers outside of the diagrams are the sum of all the induced and repressed genes under each condition. The infected fruit were inoculated with 10^4^ *P. digitatum* conidia mL^−1^ and the control mock-wounded fruit with water. After wounding or infection, all the fruit were kept at 20 °C for 1 d (1 dpi).

We also found that the number of DEGs that met a cutoff of at least a 2-fold change (−1 ≥ log2 ≥ 1) and a *p*-value of ≤0.05 induced or repressed by wounding or infection, in relation to the FH fruit, were similar and very high in Pinalate (Figure 3A) and Navelate (Figure 3B) fruit, but lower in Navelate. Most DEGs were commonly induced or repressed by wounding and infection, which resulted in a marked reduction in the number of DEGS associated specifically with infection. This effect was highlighted by a more straightforward comparison between wounding (control of infection) and infection (Figure 3C). As shown in this figure, induction prevailed over repression in Pinalate fruit, the number of DEGs induced specifically by the fungus in this cultivar was reduced by exogenous ABA, and repression prevailed in the parental Navelate cultivar. Finally, at fruit harvest, we found a large number of DEGS that were more (1344) or less (651) expressed in Pinalate than in the parental fruit (Table S3).

### 2.3. Constitutive Differences between Navelate and Pinalate Fruit

The KEGG analysis of the up- and down-regulated genes in the FH Pinalate fruit vs. the FH Navelate oranges revealed that only the down-regulated genes grouped in the differential pathways or Brite Hierarchies categories. These genes mostly included those involved in secondary metabolism, including carotenoids, phenylpropanoids and terpenoids and polyketides (Table 2), which showed large enrichment factors. This factor is the ratio between the fraction of the pathway genes in the tested set and the fraction of the pathway genes in the dataset. Moreover, the genes included abundant cytochrome 450, glycosytransferases and genes involved in plant hormone signal transduction and transport.

The TopGO analysis also revealed that the most significant BP (Table 3) and MF processes (Table S4) enriched in the FH Pinalate fruit vs. the FH parental fruit, were related to protein phosphorylation. Protein ubiquitination and the regulation of transcription were also among the most significantly-enriched BP processes in Pinalate, followed by BP related to photosynthesis and glucan metabolism and transport—including phospholipid, ammonium and ion transport (Table 3). These results agreed with the enrichment of the CC related to light harvesting photosystems, vesicle trafficking (exocyst) or membrane in Pinalate (Table 3). An examination of the MF overrepresented in the FH Pinalate fruit further highlighted the relevance of the MF involving protein kinase, DNA and ATP binding, ubiquitin–protein transferase, xyloglucan:xyloglucosyl transferase and oxidoreductase activities, and the activity of ionotropic glutamate receptors (Table S4). In this comparison, the most significant underrepresented BP were related to metabolism, oxidation–reduction and chitin catabolism, followed by BP associated with defense responses to microorganisms, glutathione metabolism or metal ion transport (Table 3). One remarkable finding was the major differences in the expression of the DEGs belonging to the UDP-glycosyltransferase and aspartyl protease family proteins, as well as of WRKY (WRKY55) and MYB (MYB68) TFs, which were much more expressed in the mutant. Another MYB TF (MYB61) showed the opposite trend and was less expressed in the mutant than in the parental cultivar (Table S5).

### 2.4. Influence of ABA in the Early Responses of Citrus Fruit to P. digitatum Infection

Most of the DEGs induced or repressed by *P. digitatum* infection in Pinalate fruit (IP vs. WP) were not differentially expressed in either their parental or the ABA-treated Pinalate fruit (Table S6). Two DEGs that encoded acyl-transferases (≈10- and 4-fold induction), a protease inhibitor protein (4.5-fold) and chlorophyllase 1 (CLH1) (3.8-fold), were the most induced. Other DEGs specifically induced in Pinalate encoded carbonic anhydrases (CAs, CA2 and ACA7), proteins involved in terpenoid metabolism—including a camelliol (triterpenoid) C synthase—an annexin (ANNAT4) and two O-methyltransferase 1 (OMTs). The induction of these DEGs was reduced in the ABA-treated Pinalate fruit and were not statistically significant (Table S6). Therefore, we cannot rule out the notion that, to some extent, exogenous ABA reversed the infection-induced up-regulation of these DEGs in the Pinalate oranges not treated with ABA. Only the gene that encoded an aspartyl protein (orange1.1g014679m.g) was significantly induced at the same level as in the Pinalate fruit treated or not with the hormone. One remarkable finding was that the two genes that encoded an ethylene response factor 1 (ERF1) (orange1.1g039409m.g and orange1.1g042174m.g) were also significantly induced in Navelate by infection, but such inductions were slightly lower in the mutant. The treatment with ABA in Pinalate also lowered the level of repressions, which partially mimicked the parental (Table S6). Such repressions were not statistically significant when the mutant was treated with ABA. Therefore, we could consider that these repressions may also be regulated by ABA in infected fruit. The number of DEGs specifically repressed in Pinalate was smaller than those induced, and the most down-regulated gene (≈8-fold repression) encoded an ATEXO (exocyst subunit exo70 family protein G1) protein. The functional categorization of these DEGs showed that they grouped in induced BP or MF, which were exclusive of this condition (Table 4, Pattern 1). The most induced MFs involved those with terpene synthase and endopeptidase inhibitor activities (Table S7). The ‘magnesium ion binding’ MF was also induced and grouped with the same terpenoid cyclases found in the ‘terpene synthase’ MF. Likewise, O-methylation, the MF processes ‘carbonate dehydratase activity’, ‘calcium-dependent phospholipid binding’ and ‘diacylglycerol O-acyltransferase activity’, and the ‘glycerolipid biosynthetic’ BP, were activated in the mutant (Table 4 and Table S7). Exogenous ABA significantly increased the expression of only three DEGS (IPA vs. WPA in Table S6) that coded for a pathogenesis-related thaumatin (ATLP-1; 3-fold), a bifunctional inhibitor/lipid-transfer protein (LTP) (2.6-fold) and a protease; the latter was significantly induced at the same level in the Pinalate fruit not treated with the hormone. Moreover, ABA-treated fruit favored the repression of five genes in Pinalate: two of unknown function, and the others which encoded a calcium-binding EF-hand, ABC transporter and oxidative stress 3 (OXS3) proteins—which accounted for the repression of calcium ion binding and the ATPase activity of MFs (Table 4, Pattern 2, Table S7).

The results found in the parental fruit (IN vs. WN) further revealed the ABA-deficient mutant’s lack of an ability to induce or repress the expression of a set of up- and down-regulated DEGs, respectively, by infection in Navelate. While they play different roles, these DEGs mostly encode stress-related proteins (Table S6). In general, this ability was barely restored by applying ABA (Table S6). Major ABA effects were observed in the repression of the DEGS that encoded OXS3, RmlC-like cupin and SAUR-like auxin-responsive proteins. However, only the repression of OXS3 was statically significant in the mutant fruit treated with ABA (Table S6). In this set of genes, it was remarkable that the two ERF1 were also induced in Pinalate. The TopGO analysis also demonstrated Pinalate’s lack of an ability to induce or repress processes that were differential in the parental in response to *P. digitatum* infection (Table 4, Pattern 3). All the differential processes induced in the infected Navelate fruit corresponded to a single gene coding for a signal recognition particle (CAO, CPSRP43) related to protein import in chloroplasts (Table S7). Likewise, the mutant was unable to repress the negatively-regulated processes in the parental, which mostly involved genes that encoded proteins with hydrolase activity. Most were also related to the cell wall and the apoplast CC, or encoded PAPs, but also coded for a GDSL-like lipase and a subtilase. Two other MFs showed nutrient reservoir and N,N-dimethylaniline monooxygenase activities, and involved an RmlC-like cupin and a flavin-binding monooxygenase protein, respectively. The RT-qPCR analysis of the DEGs (Table S1) selected for the validation of the RNA-Seq analysis was performed in the samples taken at fruit harvest and was run daily for up to 3 d (Figure S3). This period was selected to focus on early responses to infection and to discriminate others that could be associated with the development of disease symptoms. The data from both analyses agreed about the expression of most DEGs being changed by infection and wounding in both cultivars, while the effect of adding ABA to the mutant on such changes differed among DEGs. Moreover, the RT-qPCR analysis results indicated that some inductions/repressions that occurred by 1 dpi in response to infection could be transitory.

## 3. Discussion

Previous research has shown global molecular mechanisms operating in the citrus fruit-*P. digitatum* interaction and in those related to resistance elicitation against the pathogen [20,21,22,23,24,25,26], and demonstrated that ABA protects citrus fruit against *P. digitatum* [11]. However, the mechanisms by which this hormone reduces the postharvest disease are barely known [18]. To bridge this knowledge gap, we aimed to understand both the interplay between ABA and the preformed constitutive defense mechanisms related to citrus fruit resistance to *P. digitatum* and how this hormone influences the very early transcriptional outputs induced by the fungus. Therefore, by considering that disease development started by 3 dpi in Pinalate and later in its parental (Figure 1), we selected samples from both cultivars at 1 dpi and from the FH fruit for the subsequent RNA-Seq analysis. With this approach, we also discarded the stress responses associated with tissue damage that occurred because of disease development.

The analysis of the DEGs less expressed in the FH Pinalate fruit, respective to any other sample included in the RNA-Seq analysis (Cluster 12) (Table 1 and Table S2), suggested a link between ABA deficiency and the repression of a protease inhibitor (orange1.1g033055m.g) and *PR4* (orange1.1g032285m.g) DEGs, which could be related to the mutant’s lesser constitutive defenses against *P. digitatum*. In this regard, it is interesting to note that a citrus *PR4* gene has been related to elicitation of resistance in Navelate orange [21]. The Kegg and TopGO analyses further revealed constitutive differences between both cultivars harvested in the fully mature stage (Table 2 and Table 3), when differences in their susceptibility and ABA content were noticeable (Figure 1 and Figure 2). As expected, carotenoid biosynthesis was repressed in Pinalate [12]. The results also indicated that the biosynthesis of other secondary metabolites, including phenylpropanoids [20,24,25,27,28,29,30,31] and terpenoids (Table 2), related to resistance that is either natural or elicited against *P. digitatum* in citrus fruit [23,25,26,31], was repressed in the mutant. In line with this finding, it was noteworthy that although some monoterpenes may favor *P. digitatum* infection [32], many terpenoids protect plants from pathogen attacks [33], and ABA can make terpenoid levels rise in plants [34]. Likewise, ABA regulates phenylpropanoids biosynthesis during citrus fruit maturation [15] and increases their content in fruit such as berry or mango, particularly when they are exposed to light [35,36]. Accordingly, the Brite Hierarchies ‘Glycosyltransferases’ was repressed in the FH Pinalate fruit because these enzymes mediate the availability of phenylpropanoids and promote plant immune responses [37]. Another mechanism repressed in the FH Pinalate fruit was ‘plant hormone and signal transduction’, which plays a protective role against both the abiotic and biotic stresses that cause postharvest fruit losses [38,39]. Functional categorization (Table 3 and Table S4) further revealed the repression of the defense response to bacteria and fungi, including the chitinase activity, which can degrade the fungal cell wall and participate in elicitation of resistance against *P. digitatum* [31,40,41] and the OMT activity, which is relevant for the synthesis of phenylpropanoides; and of glutathione metabolism and oxidation-reduction processes, which should protect citrus fruit from the reactive oxygen species (ROS) secreted by *P. digitatum* to colonize citrus fruit [42]. In the context of this work, it is also noteworthy that the repression of glutathione metabolism may contribute to the greater susceptibility of the inner (albedo) than the outer citrus fruit peel to *P. digitatum* infection [26].

We also found a set of DEGs up-regulated in the FH Pinalate fruit in relation to the other analyzed sample (Cluster 14) (Table 1), but also to ubiquitination, phospholipid transport and, mostly, to protein binding, which were, in turn, related to disease resistance like RLPs (Table S2). RLPs are relevant for the recognition of pathogen elicitors [43], but also play other biological roles [44]. As RLPs were negatively regulated by exogenous ABA (Table S3), these genes should not play a defensive role in the citrus fruit–*P. digitatum* interaction. In line with this, we found that most of the processes induced in the FH Pinalate fruit, compared to the FH fruit of Navelate (Table 3 and Table S4), were associated with both biotic and abiotic stresses. A clear example is the induction of transmembrane transport, which involves phospholipids, because the orchestration of both phospholipid signaling and membrane trafficking is essential for plant immune responses, and may also participate in a range of signaling pathways such as cell growth, senescence and defense against abiotic stress [45]. Given that Pinalate was less resistant to infection than Navelate, we cannot rule out that, at harvest, the higher expression of the DEGs involved in these responses in the mutant than in its parental was influenced by stressful environmental factors occurring during fruit growth on trees, rather than being related to citrus fruit immunity against *P. digitatum*. However, all the genes belonging to cluster 14 were repressed by either wounding or infection in Pinalate fruit, and most were also repressed in the infected Navelate fruit. This suggests that the expression of these genes is related to a greater susceptibility to *P. digitatum* infection. Therefore, we explored the responses of both cultivars, and of the ABA-treated Pinalate fruit, to infection to better decipher the mechanisms regulated by ABA in relation to citrus fruit resistance to *P. digitatum*.

Our experimental design—used to compare the mechanisms induced by *P. digitatum* in the mutant and its parental fruit—was very restrictive because we focused on the very early responses to infection, even though we used a low conidial suspension concentration (10^4^ conidia mL^−1^) to inoculate the fruit. Therefore, the number of DEGs regulated by infection in both cultivars was small, and much smaller than that found in the peel of other citrus fruit cultivars when a much higher conidial concentration (10^6^ conidia mL^−1^) was used and RNA-Seq was performed at 3 dpi [23] rather than at 1 dpi. Under our experimental conditions, we identified a set of genes, which were specifically regulated by infection in each cultivar and in the ABA-treated mutant fruit (Table S6). We also showed that exogenous ABA could modify the infection-induced changes in the expression of many DEGs in the ABA-deficient mutant that could, thus, recover to some extent the parental transcriptional response when considering the infection stimulus (Table S6). Therefore, these DEGs should be regulated by ABA in infected fruit. By considering the protective nature of the genes/processes specifically up-regulated by infection in the mutant (Table S6 and Table 3), and that most of these responses were attenuated in both the parental and the ABA-treated mutant—which were more resistant to infection than the mutant fruit (Figure 1)—it seems reasonable to hypothesize that the induction of these responses in Pinalate would occur to cope with its greater susceptibility to *P. digitatum* infection. We cannot rule out the notion that the effect of ABA on such responses could be a consequence of its effect on reducing Pinalate susceptibility to the pathogen, but they might also be negatively modulated by ABA. The other DEGs up-regulated in Pinalate, which encoded ERF1 and an aspartyl protease, were also up-regulated in Navelate, and their expression did not increase when treating the mutant with ABA. Therefore, these DEGs were not regulated by ABA.

Of the DEGs up-regulated by infection in Pinalate fruit whose expression was reduced by ABA treatment (Table S6), we identified the genes involved in wax biosynthesis because of the induction of the gene encoding a O-acyltransferase (WSD1-like) [46], as well as in terpenoid and phenylpropanoid metabolisms. As these pathways were repressed in the mutant at fruit harvest, such specific activations could be deployed by the mutant to compensate for these metabolites’ lesser participation in its constitutive defenses compared to the parental cultivar. It is also worth highlighting the induction of genes *CA2*, *CA* and *ANNAT4*, and of kunitz protease inhibitors (*KPIs*)—which show endopeptidase inhibitor activity (Tables S6 and S7)—because they are important elements of plant defenses against pathogen infection [47,48,49]. Another remarkable finding was the induction of *CLH1,* because silencing *AtCLH1* and suppressing chlorophyll degradation [50,51] acts on the ROS balance and improve plant resistance to necrotrophic pathogens. Accordingly, of the set of genes up-regulated by infection in Pinalate, only *CLH1* induction should be associated with disease development, and this induction could be negatively regulated by ABA, which would agree with the findings showing that *CLH1* is down-regulated by ABA in plants [52] and still-green (immature) Valencia oranges are more resistant to infection by *P. digitatum* than more mature fruit [53]. Nevertheless, we should consider recent findings [54] which indicate that chlorophyll retention reduces defense against *P. italicum* in a brown orange mutant (Zong Cheng *cv.*) of the Lane Late Navel orange. This agrees with the findings of our group (unpublished results), which indicate the greater susceptibility of immature Navelina and Navelate oranges, whose peel was completely green, than that of mature fruit, in which chlorophyll has already degraded, to be infected by *P. digitatum*. These contrasting findings imply that the situation is not easy and other physiological factors related to fruit maturation or that occur in the mutant, such as loss of fruit firmness or accumulation of chlorophyll catabolic intermediates that lead to the burst of ROS [54], may influence the different role proposed for chlorophyll in citrus fruit susceptibility against necrotrophic fungi. Therefore, and considering the relevance of the above-mentioned findings and the result of the present work, the study of the role of chlorophyll in citrus fruit susceptibility to postharvest disease deserves further research. Our results also indicate that exogenous ABA favors the induction of the DEGs that encode a thaumatin PR protein (ATLP-1) and a bifunctional inhibitor/LTP in the mutant (Table S6, significant DEGs in IPA vs. WPA). As these genes are involved in defenses against pathogens in plants [55,56] and citrus fruit [23,30], they should contribute to the beneficial effect of ABA on reducing disease in Pinalate.

The herein presented results also highlight that, in citrus, ABA may be necessary for reducing the repression of some genes involved in plant defenses against pathogens. Thus we found that the repression of the DEGs down-regulated by infection in Pinalate was alleviated by ABA treatment (Table S6, significant DEGs in IP vs. WP), and that most are relevant in plant immunity. Therefore, their repression should be an early virulence mechanism induced by *P. digitatum* to colonize citrus fruit favored by the fruit ABA deficiency. These DEGs are related to cell wall and vesicle trafficking and to ribosomal proteins that are primarily involved in the translation of mRNA and lead to protein synthesis. The most repressed DEGs encoded an ATEXO protein, which was up-regulated in the parental and affects vesicle trafficking [57]. ABA deficiency also favored the repression of a Ribosomal L27 (RPL27), which is involved in translation regulation [58] and a pectin lyase (Pel) protein. In line with this, it is worth mentioning that Pels are the only pectinases capable of degrading pectin polymers directly via a β-elimination mechanism to result in the formation of 4,5-unsaturated oligogalacturonides, which are elicitors of *PR* gene expression [59]. However, the involvement of Pels in the plant–pathogen interaction seems to depend on the origin of the protein. Thus previous research by our group has shown the relevance of fungal pectin lyase on full *P. digitatum* virulence to infect citrus fruit [42]. Finally, the fact that exogenous ABA favored the repression of OXS3 in the mutant (Table S6, significant DEGs in IPA vs. WPA) was remarkable because this gene was highly repressed in the parental and its repression leads to the activation of ABA-responsive genes in plants [60].

The set of DEGs and processes significantly induced in the parental (Table 4 and Table S6, significant DEGs in IN vs. WN, and Table S7) involved the chloroplast, which is one of the main sources of ROS production, and the genes encoding H_2_O_2_-generating enzymes (GLOX and CAO). Previous research indicated that H_2_O_2_ may attack *P. digitatum*, plays a role in targeting defense genes in plants and can make the cell wall stronger against microbial enzyme attack by favoring lignification [53,61,62], a feature that has been related to the greater susceptibility of the inner than outer citrus fruit peel to *P. digitatum* infection [26]. The differences found between the parental and the mutant in the levels of the induction of these genes did not become smaller when ABA was added to the mutant. This result could be explained by the mutant’s partial insensitivity to the hormone [14,17], but also by the altered photosynthesis-related functions in the mutant reported by Romero et al. (2019) [15] when studying the role of ABA during citrus fruit maturation. Therefore, we cannot conclude that these responses are triggered by ABA, but the results of this study reinforce their relevance in citrus fruit resistance to *P. digitatum*. Other DEGs specifically up-regulated in the parental that could play a protective role against *P. digitatum* were a receptor-like protein kinase (RLK1), which is a key PRR component for the recognition of PAMPs, and the DEGs involved in phenylpropanoid metabolism (CYP75B1 flavonoid 3′-hydroxylase and UDP-glycosyltransferase) [21,28,37]. Of this set of DEGs, two encoded the ERF1 transcription factor, which is involved in eliciting resistance against *P. digitatum* [30] and in the up-regulation of lignin biosynthetic genes [63], which were also up-regulated by infection, in the ABA-deficient mutant.

Pinalate fruit also lacked diverse responses repressed by infection in its parental (Table S6, significant DEGs in IN vs. WN, and Table S7). It is interesting to note the marked repression of the gene that encoded OXS3 in the parental because the absence of nuclear OXS3s leads to the activation of ABA-responsive genes in plants [60], and the repression of this gene was the only one to be significantly favored by exogenous ABA in the mutant and, hence, an ABA-dependent response regulated by *P. digitatum* in citrus fruit. The repression of the RmlC-like cupin-encoding gene was also favored by ABA in Pinalate, although this repression was not statistically significant. Interestingly RmlC-like cupins constitute a pathogenicity factor in fungi that may counteract host defenses [64] but, unfortunately, their role in the host’s ability to cope with a pathogen attack has not yet been determined.

These overall results provide relevant information about the putative molecular mechanisms underlying the role of ABA in the protection of citrus fruit against *P. digitatum* infection, which encourage future research to further the understanding of proteomic and metabolomics events, or in the potential role of specific genes, associated with the beneficial effect of ABA in reducing postharvest rots in citrus fruits.

## 4. Materials and Methods

### 4.1. Fruit and Fungal Material

Full mature Pinalate (*a/b* external color index 0.1) and Navelate (*Citrus sinensis* (L.) Osbeck) (*a/b* external color index 0.5) oranges were harvested on the same day from the adult trees growing in the same experimental orchard at the The Spanish Citrus Germplasm Bank of the Instituto Valenciano de Investigaciones Agrarias (IVIA, Moncada, Valencia, Spain). Three replicates of 10 FH fruit of each cultivar were used to determine the color index, as previously proposed by Lafuente et al. (2014) [65], which was expressed as the *a/b* Hunter ratio. This ratio is classically used as a color index in citrus fruit [65] and is positive for orange fruit and negative when the fruit surface is mostly green. A Minolta CR-300 Chromameter (Konica Minolta Inc., USA) was used to measure the *a* and *b* values in an 8-mm measuring area at three locations around the equatorial plane of each fruit. Fruit from both cultivars free of damage were harvested and immediately delivered to the laboratory, where they were surface-sterilized by dipping fruit for 5 min in a 5% commercial bleach solution, which resulted in a final 0.19% sodium hypochlorite solution. Then, fruit were rinsed with water and dried at room temperature [19] before being assigned to different groups. Pinalate fruit were sorted into two groups containing 225 fruit each. The fruit from the first group were dipped for 2 min in a 1-mM ABA solution and those from the second group were dipped for the same period in water containing the same volume of ethanol (0.7%) used to dissolve the hormone (control fruit). This ABA concentration was selected according to previous results from our group, which proved its efficiency to reduce the disease caused by *P. digitatum* in Pinalate fruit [11]. Due to the facts explained in the Introduction, we did not examine the effects of treating Navelate fruit with ABA in the present study and, therefore, only one group of the 225 Navelate fruit was used herein. This group was also dipped in water containing 0.7% ethanol, as described above, for the control Pinalate fruit. The fruit in each group were assigned to two subgroups. The first subgroup contained three replicates of five fruit each and was used to determine the evolution of disease incidence and severity. The second subgroup included three replicates of 10 fruit each per sampling period and was employed to perform the transcriptomic analysis and to determine changes in the expression levels of the genes selected according to the transcriptomic analysis results. Half of the fruit in this subgroup were inoculated with water (mock control fruit) and the rest with a fungal conidial suspension prepared as described below. As *P. digitatum* is a wound pathogen, fruit peel was wounded with a flame-sterilized needle (4 mm depth) before inoculating them with either 10 µL of water or the conidial suspension per wound. Sixteen wounds were made in each fruit. Analyses were performed in flavedo samples because it is in this peel tissue where differences in the ABA levels between Navelate and Pinalate fruit are more evident. Only the flavedo taken from around the inoculation site was considered in the analyses. Therefore, 7-mm-diameter peel discs were taken around each inoculation site. Thus 16 peel discs were taken from each fruit and three replicates of 10 fruit per sampling period were selected in each replicate. The inner peel part (albedo) was removed with a razor blade from the peel discs and the flavedo discs in each replicate were frozen in liquid nitrogen, homogenized and kept at −80 °C for further analyses. Samples were taken at fruit harvest, and at 1, 2 and 3 d dpi in the fruit stored at 90–95% relative humidity (RH) and 20 °C.

The conidial suspension used to inoculate Navelate and Pinalate oranges, either treated or not with ABA, was prepared in sterile distilled water from isolate Pd1 (CECT 20795) [66] of the *P. digitatum* (Pers.:Fr.) Sacc fungus grown for 7 d at 24 °C on potato dextrose agar (PDA) (Thermo Fisher Scientific, Wilmington, DE, USA). The conidial concentration was determined by a hemocytometer [19] and then diluted with sterile distilled water to a final concentration of 10^4^ conidia mL^−1^ to inoculate each wound.

### 4.2. Disease Incidence Evolution and Severity Determination

Disease incidence was estimated by periodically determining the number of wounds showing disease and expressed as a percentage in relation to the total wounds inoculated with the pathogen. Four equidistant inoculations per fruit were performed on the equatorial axis of each orange and 60 wounds (three replicates of five fruit each) were considered in this analysis. Disease severity was also estimated in the same wounds by measuring the lesion diameter of each macerated zone in two perpendicular directions with a flexible ruler and was expressed as the lesion area (mm^2^). To follow disease incidence and severity, all the fruit were stored at 90–95% RH and 20 °C in plastic boxes in the dark.

### 4.3. Determination of ABA Content

ABA content was measured in flavedo by following the indirect ELISA method described by Lafuente et al. (2019) [11]. Briefly, the hormone was extracted from 200 mg of the frozen homogenized flavedo samples with 2 mL of 80% acetone containing 0.45 mM butylated hydroxytoluene and 2.5 mM citric acid in a Cell Disruptor (Mini Beadbeater 8, Biospec Products, Inc., Bartlesville, OK, USA). The supernatants obtained after centrifuging the extracts were diluted to reach ABA levels ranging within the ABA standard curve. The dilution was executed with cold TBS at pH 7.8, which was prepared with 50 mM Tris, 2 mM MgCl_2_ and 150 mM NaCl. All the diluted extracts were analyzed in duplicate in three biological replicates and ABA content was expressed on a fresh weight basis per kg flavedo.

### 4.4. Total RNA Extraction

Total RNA extraction was performed as previously described [17] from 1 g of the frozen flavedo samples. The RNA concentration was measured by a NanoDrop ND-1000 spectrophotometer (Thermo Scientific, Wilmington, DE, USA) and its integrity was confirmed by migrating RNA (1 µg) on agarose gel [17]. RNA quality was verified by using the Agilent 2100 Total RNA Bioanalyzer (Agilent Technologies, Madrid, Spain) and the RNA 6000 Nano Kit (Agilent Technologies, Madrid, Spain) before the RNA-Seq analysis.

### 4.5. RNA-Seq Analysis, Data Processing and Normalization

RNA-Seq analysis, data processing and normalization were performed as previously described by Romero and Lafuente (2020) [67]. In short, sequencing libraries were constructed from 2 μg of the RNA of the flavedo samples collected from FH Pinalate oranges, and from the Pinalate fruit treated or not with ABA, collected at 1 dpi from both fruit inoculated with *P. digitatum* and with water (mock-wounded fruit; control). RNA-Seq raw reads from the Navelate samples were retrieved from NCBI Bioproject PRJNA749665. For the Pinalate samples, three biological replicates of each sample were used. A TruSeq Stranded mRNA Library Prep Kit^®^ with PolyA selection for ribosomal RNA depletion (Illumina, San Diego, CA, USA) was utilized, and the manufacturer’s recommendations were followed. Libraries were sequenced by employing the Illumina NextSeq 500 platform, and 75-bp single-end reads were generated by the Genome Facility at the SCSIE-UV (University of Valencia, Spain, accessed on 27 September 2019). The raw sequence reads quality was examined by FastQC v0.11.8 (http://www. bioin-formatics.babraham.ac.uk) and FastP (https://doi.org/10.1093/bioinformatics/bty560, accessed on 30 September 2019). Clean data were obtained by removing the reads that contained only adaptors. Sequence reads were filtered by a mean Q20. The *Citrus sinensis* genome (Phytozome release Csinensis_154_v1.1) https://genome.jgi.doe.gov/portal/pages/dynamicOrganismDownload.jsf?organism=Phytozome, accessed on 6 March 2020) with the default settings in the TopHat2 v2.1.0 software was used to map the trimmed sequences (https://genome.jgi.doe.gov/portal/pages/dynamicOrganismDownload.jsf?organism=Phytozome, accessed on 6 March 2020). The Seqmonk v1.46 software was employed for quality control, visualization and quantification purposes (http://www.bioinformatics.babraham.ac.uk/projects/seqmonk/, accessed on 6 March 2020). The differential expression analysis between two experimental conditions was run with the edgeR R/Bioconductor package (v3.20.9) in the R (v3.4.4) environment (R Core Team, 2018). Only the genes that met an adjusted *p*-value ≤ 0.05 in each comparison were considered to be differentially expressed genes (DEGs). These values were estimated as proposed by Benjamini and Hochberg (1995) [68], and the unique gene expression levels were expressed by the Log_2_ RPM method. A cut-off of |Log2 FoldChange| ≥ 1 was established to select the DEGs to be included in the Venn diagrams, which show the number of DEGs induced or repressed by infection or wounding (mock control), and for the following bioinformatics analyses. The Seqmonk tool was employed to select highly variable genes by a standard deviation cutoff above 0.6. The selected genes were hierarchically clustered by the average linkage method with the Pearson Correlation distance metric and are represented on the heatmap and by the HCA using the MultiExperiment Viewer (MeV 4.9.0, accessed on 22 June 2020). The PCA was plotted by plot.ly (https://plot.ly, accessed on 14 September 2020) and the Log_2_ RPM values were used in the analysis. The TBtools.KeggBackEnd package (TBtools v 1.046) [69] was employed for the KEGG enrichment analysis to identify the significantly repressed or induced KEGG metabolic pathways. The TopGO (v2.30.0) package [70] with the default ‘weight01’ algorithm was employed to identify the BP, CC or MF enrichment analyses of DEGs. A GO term was only accepted as enriched if at least six DEGs were annotated for this term when the Fisher *p*-value (pgoFisher) was <0.05.

### 4.6. Analysis of Gene Expression

Total RNA was treated with Ribonuclease-free DNase (Thermo Fisher Scientific, Wilmington, DE, USA) according to the manufacturer’s instructions to remove genomic DNA contaminations for subsequent gene expression analyses. To validate the RNA-Seq results, and to determine the expression pattern of the genes selected for the validation in response to wounding (control) or infection, an RT-qPCR analysis was performed by following the method described by Romero and Lafuente (2020) [67]. cDNA was synthesized from 2 μg of total RNA from each sample by a ribonuclease inhibitor and SuperScript III RT (both from Thermo Fisher Scientific, Wilmington, DE, USA) following the manufacturer’s instructions. The DNAMAN 4.03 software (Lynnon BioSoft; https://www.lynnon.com/dnaman.html, accessed on 11 January 2021) was used to design the gene-specific forward and reverse primers, which are shown in Table S1. The normalization of the expression levels of the target genes was conducted by using the ACT and TUB genes. The expression levels of both the Navelate and Pinalate (treated or not with ABA) samples of the wounded (control) and infected fruit were referred to those found in the samples collected from the FH Navelate or Pinalate fruit, respectively, in the Relative Expression Software Tool (http://rest.gene-quantification.info, accessed on 5 October 2021). The cDNA obtained from 25 ng of RNA, the gene-specific primer pairs and SYBR Green 1 Master (Roche Diagnostics, Mannheim, Germany), were employed to obtain the relative gene expression data in a Light Cycler480 II System (Roche Diagnostics, Mannheim, Germany) instrument. cDNA amplification was monitored during 40 cycles at 95 °C (10 s), 60 °C (5 s) and 72 °C (10 s). The values were the means of three biological replicates samples, with two technical replicates ± standard error.

### 4.7. Statistical Analysis

The Statgraphics Plus 4.0 software (Manugistics, Inc., Rockville, MD, USA, accessed on 7 November 2021) was used to perform the statistical analyses. An analysis of variance (ANOVA) and a Tukey’s test (*p* < 0.05) (*p* ≤ 0.05) were run to determine if infection induced significant differences in relation to the mock-wounded samples, in Navelate fruit and in the fruit of its mutant Pinalate, either treated or not with ABA, for the same storage time. The results are the means of three biological replicated samples ± standard error.

## 5. Conclusions

To conclude, our findings provide not only new potential targets for developing *P. digitatum*-resistant varieties, but also new insights into the role of ABA in the interaction mechanisms between citrus fruit and the necrotrophic fungus *P. digitatum*. Of those targets regulated by ABA, we wish to highlight a thaumatin protein and a bifunctional inhibitor/LTP, which are both relevant in plant immunity. Moreover, the genes that play protective roles against the fungus that encoded the ATEXO, RPL27 and Pel proteins, which affect vesicle trafficking, translational regulation and favor the release of elicitors of *PR* gene expression, respectively, were repressed by infection in the ABA-deficient mutant, but not in its parental. It is also worth highlighting the *CLH1* gene, induced by infection in Pinalate, and that encoding *OXS3*, which was negatively regulated by ABA, because OXS3 family proteins repress ABA signaling in plants, and the lack of OXS3s might favor the activation of ABA-responsive genes. 

## Figures and Tables

**Table 1:** Gene ontology (GO) analysis (*p* ≤ 0.05) of the biological (BP) and molecular function (MF) processes, and of the cell components (CC), corresponding to the DEGs (*p* ≤ 0.05) belonging to clusters 12 and 14.

	GO			pgo
	Category	GO.ID	Term	Fisher
**Cluster 12**	BP	GO:0050832	defense response to fungus	1.50 × 10^3^
	BP	GO:0042742	defense response to bacterium	2.00 × 10^3^
	BP	GO:0009611	response to wounding	4.40 × 10^3^
	MF	GO:0016747	acyltransferase activity, transferring groups other than amino-acyl groups	3.10 × 10^3^
	MF	GO:0004867	serine-type endopeptidase inhibitor activity	3.40 × 10^3^
	CC	None		
**Cluster 14**	BP	GO:0016567	protein ubiquitination	8.50 × 10^3^
	BP	GO:0015914	phospholipid transport	1.75 × 10^3^
	MF	GO:0004842	ubiquitin-protein transferase activity	9.60 × 10^3^
	MF	GO:0004402	histone acetyltransferase activity	1.25 × 10^3^
	MF	GO:0005515	protein binding	1.47 × 10^2^
	MF	GO:0004012	phospholipid-translocating ATPase activity	1.62 × 10^2^
	CC	None		

**Table 2:** Metabolic pathways and Brite Hierarchies enriched (UP) or under-represented (DOWMN) by the KEGG analysis in the freshly-harvested (FH) Pinalate fruit vs. the FH Navelate fruit. Three biological replicates from each condition were used. Only the DEGs (*p* ≤ 0.05) that met a cutoff of |Log_2_ FoldChange| ≥ 1 were considered in the analysis.

	Term Name	MainClass	Enrich	Corrected *p*-Value
			Factor	(BH Method)
UP	None			
DOWN	A09100 Metabolism	A09100 Metabolism	1.56	3.23 × 10^4^
	B09109 Metabolism of terpenoids and polyketides	A09100 Metabolism	3.36	2.05 × 10^3^
	00199 Cytochrome P450	A09180 Brite Hierarchies	5.81	6.47 × 10^3^
	00908 Zeatin biosynthesis	A09100 Metabolism	7.66	1.14 ×x 10^2^
	04075 Plant hormone signal transduction	A09130 Environmental Information Processing	2.54	1.20 × 10^2^
	01003 Glycosyltransferases	A09180 Brite Hierarchies	3.01	1.16 × 10^2^
	00906 Carotenoid biosynthesis	A09100 Metabolism	6.22	1.76 × 10^2^
	00940 Phenylpropanoid biosynthesis	A09100 Metabolism	2.85	3.31 × 10^2^
	02000 Transporters	A09180 Brite Hierarchies	1.91	3.50 × 10^2^

**Table 3:** Gene ontology (GO) analysis (*p* ≤ 0.05) of the biological processes (BP) and cellular components (CC) up- (Up) or down-regulated (Down) in the flavedo of the freshly-harvested (FH) Pinalate fruit vs. the FH parental fruit. Only the DEGs (*p* ≤ 0.05) that met a cutoff of |Log_2_ FoldChange| ≥ 1 were considered in the analysis. Three biological replicates from each condition were used.

	Go			pgo
	Category	GO.ID	Term	Fisher
Up	BP	GO:0006468	protein phosphorylation	2.90 × 10^17^
	BP	GO:0016567	protein ubiquitination	3.60 × 10^5^
	BP	GO:0006355	regulation of transcription, DNA-templat…	5.40 × 10^5^
	BP	GO:0048544	recognition of pollen	2.00 × 10^3^
	BP	GO:0009765	photosynthesis, light harvesting	3.00 × 10^3^
	BP	GO:0006073	cellular glucan metabolic process	1.10 × 10^2^
	BP	GO:0015914	phospholipid transport	2.20 × 10^2^
	BP	GO:0072488	ammonium transmembrane transport	3.90 × 10^2^
	BP	GO:0006811	ion transport	3.90 × 10^2^
	BP	GO:0015979	photosynthesis	4.40 × 10^2^
	CC	GO:0048046	apoplast	2.00 × 10^3^
	CC	GO:0000145	exocyst	4.30 × 10^3^
	CC	GO:0016021	integral component of membrane	1.15 × 10^2^
	CC	GO:0009522	photosystem I	2.34 × 10^2^
	CC	GO:0009523	photosystem II	2.36 × 10^2^
Down	BP	GO:0008152	metabolic process	4.70 × 10^8^
	BP	GO:0055114	oxidation-reduction process	2.40 × 10^6^
	BP	GO:0006032	chitin catabolic process	8.70 × 10^5^
	BP	GO:0016998	cell wall macromolecule catabolic process	1.20 × 10^4^
	BP	GO:0050832	defense response to fungus	3.20 × 10^3^
	BP	GO:0006749	glutathione metabolic process	6.26 × 10^3^
	BP	GO:0042742	defense response to bacterium	6.26 × 10^3^
	BP	GO:0048573	photoperiodism, flowering	6.26 × 10^3^
	BP	GO:0009909	regulation of flower development	1.50 × 10^2^
	BP	GO:0030001	metal ion transport	2.74 × 10^2^
UP/	CC	GO:0016020	membrane	5.90 × 10^7^
Down				1.00 × 10^5^

**Table 4:** Comparison of the biological (BP) and molecular function (MF) processes, and of the cell components (CC) induced (↑) or repressed (↓), in the Pinalate fruit treated or not with ABA, and in the Navelate fruit inoculated with *P. digitatum* (10^4^ conidia mL^−1^) by 1 d post-inoculation (1 dpi) vs. their wounded (control) fruit. Only the DEGs (*p* ≤ 0.05) that met a cutoff of |Log_2_ FoldChange| ≥ 1 were considered in the analysis. Three biological replicates from each condition were used.

GO				pgo		pgo
Category	GO ID	Term	Up	Fisher	Down	Fisher
**Pattern 1: Regulated by *P. digitatum* in Pinalate**						
BP	GO:0045017	glycerolipid biosynthetic process	↑	2.50 × 10^2^		
MF	GO:0010333	terpene synthase activity	↑	1.10 × 10^4^		
MF	GO:0004866	endopeptidase inhibitor activity	↑	1.03 × 10^3^		
MF	GO:0008171	O-methyltransferase activity	↑	5.92 × 10^3^		
MF	GO:0004089	carbonate dehydratase activity	↑	7.27 × 10^3^		
MF	GO:0000287	magnesium ion binding	↑	1.00 × 10^2^		
MF	GO:0005544	calcium-dependent phospholipid binding	↑	1.31 × 10^2^		
MF	GO:0004144	diacylglycerol O-acyltransferase activit…	↑	1.74 × 10^2^		
CC	None		↑			
BP	None				↓	
MF	None				↓	
CC	None				↓	
**Pattern 2: Regulated by *P. digitatum* in Pinalate fruit treated with ABA**						
BP	None		↑			
MF	None		↑			
CC	None		↑			
BP	None				↓	
MF	GO:0005509	calcium ion binding			↓	1.30 × 10^2^
MF	GO:0016887	ATPase activity			↓	3.40 × 10^2^
CC	None				↓	
**Pattern 3: Regulated by *P. digitatum* in Navelate**						
BP	GO:0045038	protein import into chloroplast thylakoi.	↑	7.40 × 10^4^		
BP	GO:0009416	response to light stimulus	↑	8.06 × 10^3^		
MF	None		↑			
CC	GO:0080085	signal recognition particle, chloroplast..	↑	3.40 × 10^4^		
CC	GO:0009507	chloroplast	↑	2.35 × 10^3^		
BP	GO:0006073	cellular glucan metabolic process			↓	1.20 × 10^3^
MF	GO:0016762	xyloglucan:xyloglucosyl transferase acti..			↓	4.00 × 10^4^
MF	GO:0004499	N,N-dimethylaniline monooxygenase activi..			↓	1.11 × 10^2^
MF	GO:0045735	nutrient reservoir activity			↓	1.11 × 10^2^
MF	GO:0016787	hydrolase activity			↓	1.13 × 10^2^
MF	GO:0050661	NADP binding			↓	3.30 × 10^2^
MF	GO:0004553	hydrolase activity, hydrolyzing O-glycos..			↓	4.88 × 10^2^
CC	GO:0048046	apoplast			↓	2.40 × 10^4^
CC	GO:0005618	cell wall			↓	2.31 × 10^3^

## Data Availability

Data supporting the findings of this study are available in this publication and its Supplementary Materials published online. The datasets used for the transcriptomic study can be found at the NCBI repository (BioProject ID PRJNA892523, https://www.ncbi.nlm.nih.gov/bioproject/PRJNA892523).

## Supplementary Materials

The following supporting information can be downloaded at: https://www.mdpi.com/article/10.3390/ijms232415796/s1.
