# Supplementary material for: The Role of ABA in the Interaction between Citrus Fruit and Penicillium digitatum
Source: Int J Mol Sci. 2022 Dec 13;23(24):15796. doi: 10.3390/ijms232415796 (PMC9779756; doi:10.3390/ijms232415796)
Supplement: Supplementary file 1 [file ijms-23-15796-s001.zip › Certificado Revisi¿«n T Lafuente.pdf]

h y a

Helen L. Warburton
Marqués de Caro, 11-3ª
46003 VALENCIA

Tax. No. X0848511H
Tel:+34 605 344 082
Email: hya@telefonica.net

(English translations and corrections of English texts)

26 October 2022

Dear sir or madam,

I, Helen Warburton, native English writer, am registered in Inland Revenue's professional Epigraph 774 in the city of Valencia, since 28th October, 2003, as a translator and English language editor. I am also registered in several Spanish universities and Spanish research institutes to provide my translating and English language editing services. I am registered with the Spanish Data Protection Agency (AEPD). I currently work with the following research centres and firms in Spain:

UA, UCLM, ULPGC, UNIZAR, UJI, UMH, UMU, UNED, UPC, UPCT, UPV, UV, el Complejo Hospitalario de Albacete, IATA, IBMCP, IMDEA, INIA, IVI, IVIA, ASEPUC, CIBER, CIPF, CEAM, ESPAITEC, FISABIO, FULP, SESCAM, Fund. SECT, Fund. Hospital La Fe, Fund. Parc Científic UV, Univ. Federal do Recôncavo da Bahia, Univ. Federal de Viçosa, CEU San Pablo, Jabones Beltrán, Avanqua, etc.

I have recently checked and edited the article presented in English, written by María T. Lafuente and Luis González-Candelas, entitled:

The role of ABA in the interaction between citrus fruit and *Penicillium digitatum*

TRADUCCIONES - REVISIONES
Helen Warburton
NIF: X08148511H
Marqués de Caro, 11-3
46003 - VALENCIA - 605 344 082

Yours faithfully

Helen L. Warburton

This document contains confidential information, whose content is protected by law. Anyone other than the addressee cannot reproduce, use or diffuse this complete or partial content. If you receive this by mistake, please notify the sender immediately for the necessary steps to be taken.
