# Supplementary material for: The Role of ABA in the Interaction between Citrus Fruit and Penicillium digitatum
Source: Int J Mol Sci. 2022 Dec 13;23(24):15796. doi: 10.3390/ijms232415796 (PMC9779756; doi:10.3390/ijms232415796)

Supplementary Materials: The following supporting information can be downloaded at: https://www.mdpi.com/article/10.3390/ijms232415796/s1

Table S1: The primers and genes selected for the RT-qPCR analysis

Gene	Citrus ID	Description	Forward (5' - 3')	Reverse (5' - 3')
CsPeL	*orange1.1g009075m.g*	pectin lyase-like superfamily protein	GTGACCCATTTACAGGTATT	ACAGGCTCTTATTTTCTCAG
CsCLH1	*orange1.1g020188m.g*	chlorophyllase 1	TCAAGACAGTGAATAAACCA	AGATTGGAATAGCTTGTGTT
CsACA7	*orange1.1g039445m.g*	alpha carbonic anhydrase 7	TGGAAGACCTGATTCTCTAT	CGTAAGTTTATTGGTTGTTG
CsCAMS1	*orange1.1g006042m.g*	camelliol C synthase 1	ATCTCTACTATCCCCATCCT	GTCCTCGTAATGAATGAACT
CsANNAT4	*orange1.1g021607m*	annexin 4	GCTTACCATTCTCTCTTTGA	GCTTGCTCCTTGTAGATAGT
CsGLOX	*orange1.1g048017m*	glyoxal oxidase-related protein	TGTTACCTTCTGGTGATGTA	TACTCCCACCCACTAATATC
CsCSLB04	*orange1.1g038469m.g*	cellulose synthase-like B4	TCAGACTACATTCGTATTGG	CATTTTATCATCGTCTTCG
CsCYS1	*orange1.1g021775m.g*	Cysteine Synthase C1 (β-Cyanoalanine Synthase)	GCAGCGAACCCATGTTGATGAG	GGCCACTGCTGGTCTGTCC
CsACT	orange1.1g037845m.g	*Actin*	TTAACCCCAAGGCCAACAGA	TCCCTCATAGATTGGTACAGTATGAGA
CsTUB	orange1.1g036688m.g	*Tubulin*	GCATCTTGAACCCGGTAC	ATCAATTCGGCGCCTTCAG

Table S2: The identification of the DEGs included in each biological process (BP) and molecular function (MF) (see Table 1) belonging to clusters 12 and 14.

See excel

Table S3: The global data obtained by RNA-Seq analysis.

See excel

Table S4: The gene ontology (GO) analysis (P ≤ 0.05) of the molecular function (MF) processes enriched (Up) or under-represented (Down) in the flavedo of the freshly harvested (FH) Pinalate fruit in relation to the FH parental fruit. Only the DEGs (*p* ≤ 0.05) that met a cutoff of |Log_2 FoldChange| ≥ 1 were considered in the analysis. Three biological replicates from each condition were used

	GO.ID	Term	pgo Fisher
UP	GO:0004672	protein kinase activity	1.20E-18
	GO:0003700	DNA binding transcription factor activit...	2.50E-10
	GO:0030247	polysaccharide binding	6.30E-06
	GO:0043565	sequence-specific DNA binding	3.20E-05
	GO:0005524	ATP binding	3.50E-05
	GO:0004842	ubiquitin-protein transferase activity	9.90E-05
	GO:0003849	3-deoxy-7-phosphoheptulonate synthase ac...	1.10E-03
	GO:0016702	oxidoreductase activity, acting on singl...	4.10E-03
	GO:0016762	xyloglucan:xyloglucosyl transferase acti...	7.60E-03
	GO:0005516	calmodulin binding	1.09E-02
	GO:0004674	protein serine/threonine kinase activity	1.11E-02
	GO:0042132	fructose 1,6-bisphosphate 1-phosphatase ...	1.27E-02
	GO:0004012	phospholipid-translocating ATPase activi...	1.83E-02
	GO:0017025	TBP-class protein binding	2.43E-02
	GO:0008519	ammonium transmembrane transporter activ...	3.25E-02
	GO:0031683	G-protein beta/gamma-subunit complex bin...	3.88E-02
	GO:0019001	guanyl nucleotide binding	3.97E-02
	GO:0004970	ionotropic glutamate receptor activity	4.73E-02
Down	GO:0016758	transferase activity, transferring hexos...	4.50E-11
	GO:0005506	iron ion binding	6.60E-09
	GO:0016705	oxidoreductase activity, acting on paire...	7.30E-08
	GO:0020037	heme binding	2.10E-07
	GO:0004568	chitinase activity	5.10E-05
	GO:0016747	transferase activity, transferring acyl ...	1.00E-04
	GO:0004144	diacylglycerol O-acyltransferase activit...	4.60E-03
	GO:0008429	phosphatidylethanolamine binding	5.00E-03
	GO:0003700	DNA binding transcription factor activit...	1.25E-02
	GO:0046873	metal ion transmembrane transporter acti...	1.53E-02
	GO:0045300	acyl-[acyl-carrier-protein] desaturase a...	1.64E-02
	GO:0004867	serine-type endopeptidase inhibitor acti...	2.15E-02
	GO:0005544	calcium-dependent phospholipid binding	2.71E-02
	GO:0008171	O-methyltransferase activity	3.03E-02
	GO:0004722	protein serine/threonine phosphatase act...	3.54E-02
	GO:0050660	flavin adenine dinucleotide binding	4.50E-02
	GO:0016491	oxidoreductase activity	4.69E-02
UP/ Down	GO:0005215	transporter activity	1.10E-02<br>1.65E-02

Table S5: The constitutive differences between Navelate and Pinalate fruit. The DEGs that met a *p*-adjusted value ≤ 0.05 and a cut-off of |Log_2 Fold Change| ≥ 1 in the comparison of the freshly harvested (FH) Pinalate and Navelate fruit. The fold change expression (FC) and Log_2 FC values of the compared conditions are included. Positive numbers denote higher expression values in the first term of the comparison (Pinalate). Negative values mean higher expression values in the second term (Navelate).

See excel

Table S6: The DEGs that met a *p*-adjusted value ≤ 0.05 and a cut-off of |Log2 Fold Change| ≥ 1 in the specific comparison indicated and highlighted in yellow (IP vs. WP, infection Pinalate vs. wounding Pinalate), green (IPA vs. WPA, infection ABA-treated Pinalate vs. wounding ABA-treated Pinalate) and orange (IN vs. WN, infection Navelate vs. wounding Navelate). The fold change expression (FC) and Log_2 FC values of the compared conditions are included. Positive numbers denote higher expression values in the first term of the comparison. Negative values mean higher expression values in the second term.

See excel

Table S7: Identification of the DEGs (*p*-adjusted value ≤ 0.05 and a cut-off of |Log_2 Fold Change| ≥ 1) included in each induced or repressed biological process (BP), molecular function (MF) and cellular component (CC) (see Table 4) in the flavedo of the infected Pinalate oranges samples (treated or not with ABA) and of the infected fruit of its parental Navelate in relation to their control wounded samples.

See excel

Figure S1: Multiple linear regression analysis (R2) for the comparisons made between the RT-qPCR gene expression and the RNA-Seq data for the DEGs included in Table S1. The expression of these DEGs was quantified in the freshly harvested Navelate and Pinalate fruit, and in the wounded and infected Navelate, Pinalate and ABA-treated Pinalte fruit taken from fruit at 1 dpi.

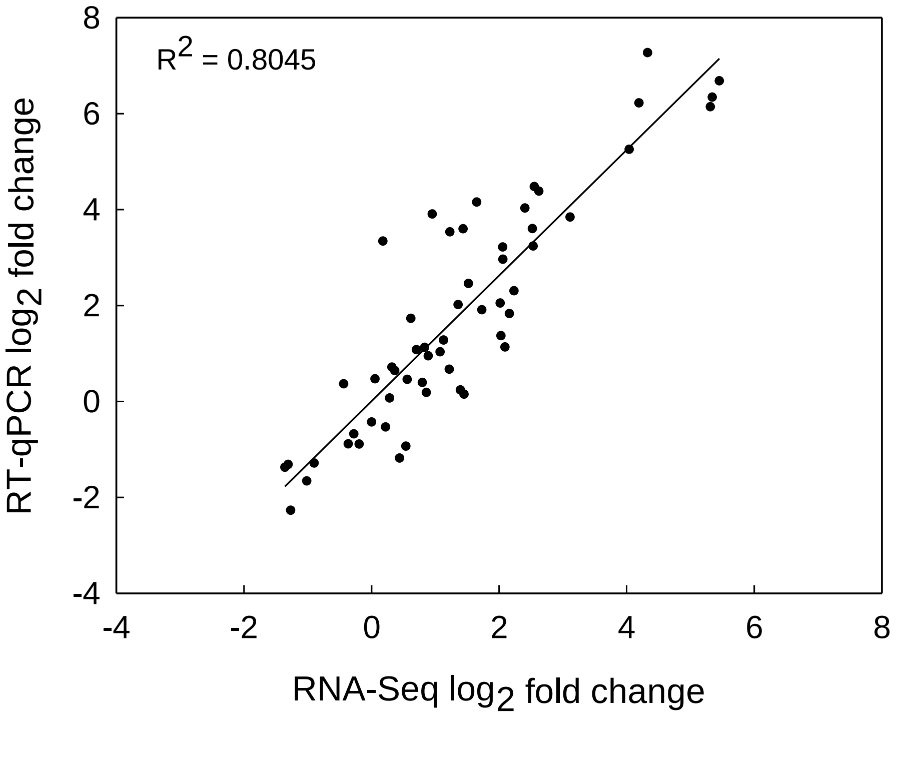

Figure S2: The 19 gene clusters identified after the gene clustering analysis of the 615 DEGs found in all the comparisons represented on the heatmap (see Figure 2C).

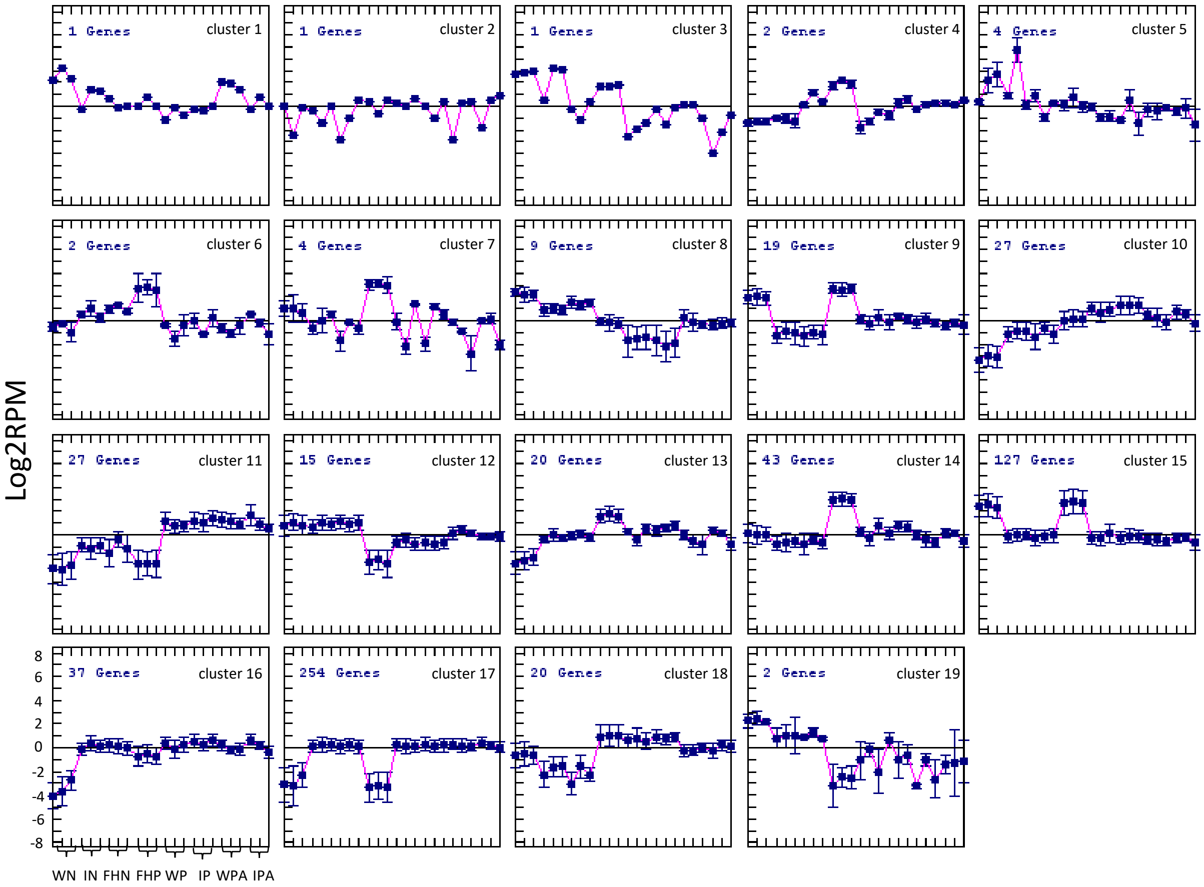

Figure S3: Relative gene expression of the selected DEGs (see Table S1) in the flavedo of the wounded (○) and infected (●) Pinalate fruit, treated or not with ABA, and of the fruit of its parental, in relation to the freshly harvested Pinalate or Navelate fruit. Changes in expression were determined in the fruit inoculated with *P. digitatum* (10^4 conidia mL^{-1}) or water (control), and left in the dark for up to 3 d at 20 ºC. The error interval indicates the standard error of the determined mean value. * denotes significant differences ($p \leq 0.05$) between the infected and the control wounded samples for the same storage time.

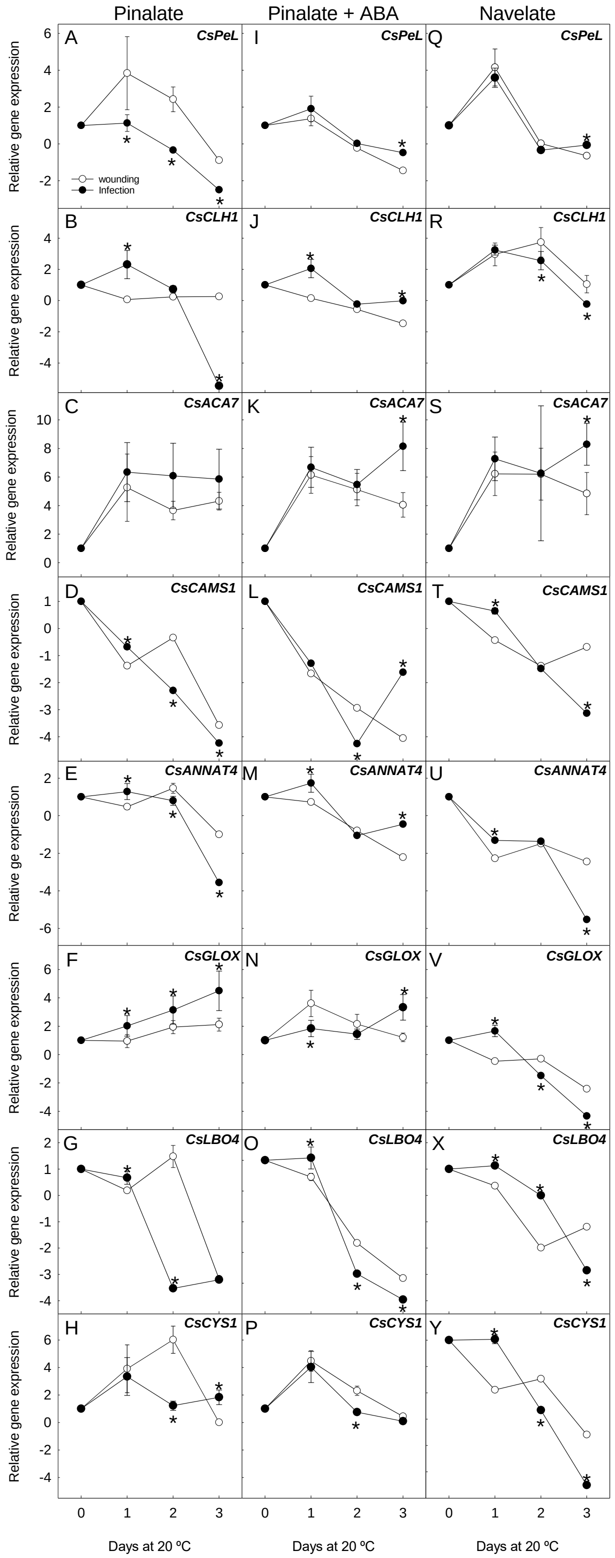

Supplement: Supplementary file 1 [file ijms-23-15796-s001.zip › IJMS_SupplemMat.pdf]
